# A novel cell-based immunofluorescence assay for the detection of autoantibodies to myelin-associated glycoprotein

**DOI:** 10.3389/fneur.2023.1289810

**Published:** 2023-12-14

**Authors:** Sara Mariotto, Piera de Gaspari, Dominik Jäger, Stefanie Hahn, Cindy Forni, Sandra Saschenbrecker, Erik Lattwein, Alessandro Dinoto, Sergio Ferrari

**Affiliations:** ^1^Neurology Unit, Department of Neurosciences, Biomedicine and Movement Sciences, University of Verona, Verona, Italy; ^2^Neuroimmunology Group, Pediatric Research Institute, Padua, Italy; ^3^Institute for Experimental Immunology, affiliated to EUROIMMUN Medizinische Labordiagnostika AG, Lübeck, Germany

**Keywords:** cell-based assay, HNK-1, human natural killer-1, IgM autoantibodies, MAG, myelin-associated glycoprotein, neuropathy

## Abstract

Peripheral neuropathy with antibodies to myelin-associated glycoprotein (MAG) is an autoimmune demyelinating disorder of the peripheral nervous system caused by pathogenic IgM recognizing the human natural killer-1 glycoepitope expressed on MAG. This study aimed to analyze the performance of a new indirect immunofluorescence cell-based assay (CBA, EUROIMMUN) for the detection of anti-MAG IgM. Antibody reactivity was determined in sera from 95 patients with clinical and neurophysiological evidence of anti-MAG-associated neuropathy and in control samples from 55 patients with other forms of peripheral neuropathy. Compared to the results of the gold standard method (ELISA, Bühlmann) and using samples at a dilution of 1:100, the CBA had a sensitivity of 98.9% and a specificity of 100% (PPV 100%, NPV 98.2%). In conclusion, the CBA allows the detection of antibodies to MAG using an easy and standardized technique, and it presents a sensitive and specific alternative to the more time-consuming ELISA. Larger studies are needed to address anti-MAG titer monitoring in parallel with clinical activity.

## 1 Introduction

Myelin-associated glycoprotein (MAG) is a 100 kDa transmembrane glycoprotein expressed by Schwann cells and represents a target of monoclonal IgM autoantibodies (MAG-Abs). These antibodies recognize the human natural killer-1 (HNK-1) carbohydrate epitope, which is highly expressed on MAG. MAG-Abs are associated with a slowly progressive large-fiber distal symmetrical sensorimotor polyneuropathy with prominent sensory impairment, ataxia, and intention tremor (1–5). The pathogenic role of MAG-Abs has been confirmed by pathological studies on nerve biopsies, showing a relationship between the widening of myelin lamellae (with deposits of IgM M-protein and complement) and segmental demyelination (6–8). Antibodies to MAG can be detected in patient sera by enzyme-linked immunosorbent assay (ELISA) or Western blot using MAG or purified myelin (9–11). For both methods of MAG-Abs detection, a normal range should be defined, as low titers may be found in normal subjects (12). ELISA is often preferred to Western blot because it is more sensitive in detecting serum MAG-Abs, easier to use, and allows antibody titration, which is potentially useful for monitoring responses to therapies (13).

The aim of this study was to determine the clinical performance of a novel immunofluorescence fixed cell-based assay (CBA, EUROIMMUN Medizinische Labordiagnostika AG, Lübeck, Germany) in comparison with the gold-standard technique used to identify MAG-Abs (ELISA, Bühlmann, Schönenbuch, Switzerland).

## 2 Patients and methods

### 2.1 Patients

Sera of consecutive MAG-Abs-positive subjects (n = 95) with a compatible clinical and electrophysiological phenotype diagnosed between August, 2018 and March, 2021 at the Neuropathology Laboratory, University of Verona, Italy were analyzed using the novel fixed CBA. A group of MAG-Abs-negative subjects with peripheral neuropathy of a different origin (n = 55) was also tested for comparison.

### 2.2 MAG-Abs ELISA

This assay (Bühlmann) employs a quantitative enzymatically amplified sandwich-type immunoassay technique. Plates precoated with highly purified MAG from human brain were used. Briefly, reconstituted calibrators and diluted patient sera were incubated for 2 h in wells, and MAG-Abs were bound by the immobilized human MAG. After washing steps, horseradish peroxidase-labeled antibodies to human IgM were added to the wells and incubated for 2 h. After washing steps, the substrate solution containing tetramethylbenzidine was added and incubated for 30 min. The proportion of MAG-Abs was revealed in a blue scale. The color development was stopped by adding H_2_SO_4,_ which caused the blue solution to turn yellow. The intensity of the color absorbance was measured in a microplate reader at a wavelength of 450 nm. The absorbance measured was directly proportional to the concentration of MAG-Abs. The concentration of MAG-Abs in samples was calculated in comparison to the standard curve, plotted with human MAG-Abs calibrators, and expressed as Bühlmann titer units (BTU).

### 2.3 MAG-Abs fixed CBA

The EUROIMMUN Anti-MAG IgM CBA is a research-use-only indirect immunofluorescence test. Each test field on the slides contains two BIOCHIPs, one coated with recombinant HEK-293 cells expressing the MAG/HNK-1 antigen and the other coated with control-transfected HEK-293 cells. In brief, samples (diluted from 1:10 to 1:1,000,000) were incubated with the test substrates for 30 min at room temperature. After washing, the slides were incubated with fluorescein isothiocyanate (FITC)-labeled goat anti-human IgM (EUROIMMUN) for 30 min at room temperature. After washing and embedding, the slides were evaluated by fluorescence microscopy. Samples were classified as positive or negative based on the fluorescence intensity of the MAG/HNK-1-expressing cells in direct comparison with control-transfected cells and control samples. Endpoint titers refer to the highest dilution showing visible fluorescence.

### 2.4 Statistics

Data are presented as median (range) and number (percentage), as appropriate. Sensitivity, specificity, positive predictive values (PPV) and negative predictive values (NPV) were calculated. Confidence intervals (95% CI) were calculated according to the Clopper-Pearson method. Sensitivity, specificity, PPV and NPV were calculated for the CBA at 1:10 and 1:100 dilution. Lastly, the correlation between titers obtained by ELISA and CBA was evaluated with a two-tailed Pearson’s correlation analysis.

## 3 Results

**Figure 1:**
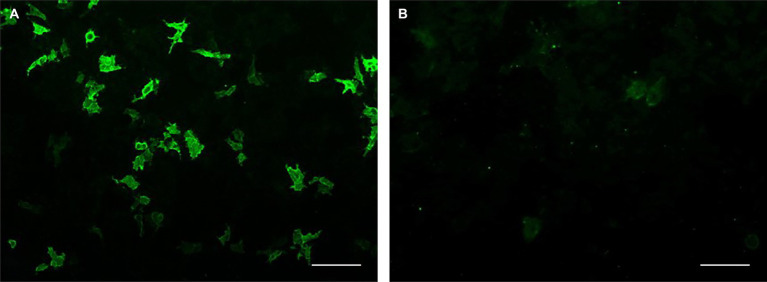
Recombinant cell-based indirect immunofluorescence assay. Serum from a patient with anti-MAG neuropathy was incubated with **(A)** HEK-293 cells expressing recombinant MAG/HNK-1 antigen and **(B)** control-transfected HEK-293 cells. Scale bar: 100 μm.

We analyzed 95 patients (*F* = 32, median age 74 years, range 49–92) with clinical and neurophysiological signs compatible with anti-MAG-associated neuropathy confirmed using the recommended ELISA (>1,000 BTU). All were positive at a 1:10 titration using EUROIMMUN’s fixed CBA. When performing further titrations, 94/95 were also positive at a 1:100 dilution, 74 at 1:1,000, 60 at 1:10,000, 20 at 1:100,000, and 11 at 1:1,000,000 (Figure 1).

Among cases with “weak positivity” (n = 33) according to the standardized ELISA technique (1:1,000–1:10,000 BTU), 28 had a titer of ≤1:1,000 using the fixed CBA (1:10 n = 1, 1:100 n = 18, 1:1,000 n = 9). Among cases with “strong positivity” (n = 62) according to the standardized ELISA technique (≥1:10,000 BTU), 55 had a titer ≥1:10,000 using the fixed CBA (1:10,000 n = 38, 1:100,000 n = 7, 1:1,000,000 n = 10).

All patients with other forms of peripheral neuropathy, included as controls (n = 55, *F* = 18, median age 67 years, range 7–91), were negative by ELISA. Of these, 6 were positive by CBA at a 1:10 dilution and none was positive at 1:100 or further titrations.

When comparing results of the fixed CBA with those of the reference ELISA technique, we observed that at a 1:10 dilution, the CBA had 100% (CI 96.2–100%) sensitivity and 89.1% specificity (CI 77.8–95.9%, PPV 94.1%, NPV 100%), while at a 1:100 dilution, the sensitivity was 98.9% (CI 94.3–100%) and the specificity 100% (CI 93.5–100%, PPV 100%, NPV 98.2%). There was a trend toward correlation between the titer observed using the two techniques (*p* = 0.07).

## 4 Discussion

We herein provide preliminary comparative data of MAG-Abs testing using the recommended ELISA technique and a novel ready-to-use fixed CBA. For the assessment of anti-MAG positivity, our results support the use of the novel CBA at a dilution of 1:100, which has the highest specificity (100%) and a very good sensitivity (98.9%). Compared to the other methods, this easy technique has many advantages, including [i] the expression of natively folded antigen in the membranes of the cell substrate enabling the detection of Abs binding to conformational epitopes, [ii] flexibility in assay design (BIOCHIP mosaics), and [iii] fluorescence stability, which allows multiple persons to analyze the slides and exchange images among centers. This standardized technique minimizes inter-assay variability and enables efficient preparation of large, standardized batches in a short time. In addition, it allows both identification and quantification (using further titrations) of specific antibodies.

Italian guidelines for MAG-Abs testing were recently developed through a consensus process that also reported indications and limits of anti-MAG testing, instructions for results interpretation, and an agreed-upon laboratory protocol (14). This consensus supports the use of ELISA as the most appropriate diagnostic technique, although the best cut-off of positivity is still an open question. In accordance with a previous study (13), sera with values between 1,000 and 10,000 BTU are interpreted as “weak positive” and are prone to diagnostic issues. Furthermore, a recent study identified a grey area between 1,500 and 7,000 BTU in which the differential diagnosis between chronic inflammatory demyelinating polyneuropathy (CIDP) and anti-MAG-associated neuropathy is challenging (15). In this scenario, CBA allows monitoring of MAG-Abs titer levels through further titrations, which is important for clinical correlations (16). In addition, fluorescent CBA is based on an easy and standardized technique, that is already used by many laboratories to identify antibodies to central nervous system antigens, such as antibodies to neuronal surface antigens in patients with autoimmune encephalitis or to aquaporin-4 in subjects with neuromyelitis optica spectrum disorders (17). Although qualitative concordance between CBA and ELISA results is promising, titers and BTUs are not perfectly comparable. Specific studies examining MAG-Abs titers in relation with clinical activity will clarify the suitability of this novel assay for disease monitoring.

In conclusion, the novel immunofluorescence CBA analyzed herein has a good sensitivity/specificity for the detection of MAG-Abs and retains several practical advantages. Larger studies designed with the aim of comparing and monitoring MAG-Abs titers in parallel with clinical activity are mandatory to confirm and expand our data.

## Data availability statement

The raw data supporting the conclusions of this article will be made available by the authors, without undue reservation.

## Ethics statement

Ethical approval was not required for the studies involving humans because only leftover samples after completion of all diagnostic measures were included and patient data were processed anonymously. The studies were conducted in accordance with the local legislation and institutional requirements. Written informed consent for participation was not required from the participants or the participants’ legal guardians/next of kin in accordance with the national legislation and institutional requirements because only leftover samples after completion of all diagnostic measures were included and patient data were processed anonymously. All samples analyzed are part of Biob-Neu-DNA-2014.

## Author contributions

SM: Data curation, Formal analysis, Investigation, Methodology, Writing – original draft, Writing – review & editing. PG: Data curation, Investigation, Methodology, Writing – review & editing. DJ: Conceptualization, Supervision, Writing – review & editing. SH: Conceptualization, Methodology, Validation, Writing – review & editing. CF: Methodology, Validation, Writing – review & editing, Conceptualization. SS: Writing – review & editing. EL: Conceptualization, Methodology, Validation, Writing – review & editing. AD: Data curation, Formal analysis, Investigation, Writing – review & editing. SF: Conceptualization, Project administration, Supervision, Writing – review & editing.
